# miR-497-5p inhibits cell proliferation and invasion by targeting KCa3.1 in angiosarcoma

**DOI:** 10.18632/oncotarget.11252

**Published:** 2016-08-12

**Authors:** Yaobing Chen, Dong Kuang, Xia Zhao, Dong Chen, Xiaoyan Wang, Qin Yang, Jie Wan, Yuanli Zhu, Yu Wang, Shiying Zhang, Ying Wang, Qiang Tang, Mikio Masuzawa, Guoping Wang, Yaqi Duan

**Affiliations:** ^1^ Institute of Pathology, Tongji Hospital, Tongji Medical College, Huazhong University of Science and Technology, Wuhan 430030, China; ^2^ Department of Pathology, School of Basic Medicine, Tongji Medical College, Huazhong University of Science and Technology, Wuhan 430030, China; ^3^ Department of Pharmacology, School of Basic Medicine, Tongji Medical College, Huazhong University of Science and Technology, Wuhan 430030, China; ^4^ Department of Regulation Biochemistry, Kitasato University School of Allied Health Sciences, Minamiku, Sagamihara Kanagawa, 252-0329, Japan

**Keywords:** sarcoma, cell cycle, potassium channel, hemangioma

## Abstract

Angiosarcoma is a rare malignant mesenchymal tumor with poor prognosis. We aimed to identify malignancy-associated miRNAs and their target genes, and explore biological functions of miRNA and its target in angiosarcoma. By miRNA microarrays and reverse transcription polymerase chain reaction, we identified 1 up-regulated miRNA (miR-222-3p) and 3 down-regulated miRNAs (miR-497-5p, miR-378-3p and miR-483-5p) in human angiosarcomas compared with human capillary hemangiomas. The intermediate-conductance calcium activated potassium channel KCa3.1 was one of the putative target genes of miR-497-5p, and marked up-regulation of KCa3.1 was detected in angiosarcoma biopsy specimens by immunohistochemistry. The inverse correlation of miR-497-5p and KCa3.1 also was observed in the ISO-HAS angiosarcoma cell line at the mRNA and protein levels. The direct targeting of KCa3.1 by miR-497-5p was evidenced by reduced luciferase activity due to complementary binding of miR-497-5p to KCa3.1 mRNA 3′ untranslated region. For the functional role of miR-497-5p/KCa3.1 pair, we showed that application of TRAM-34, a specific KCa3.1 channel blocker, or transfection of ISO-HAS cells with KCa3.1 siRNA or miR-497-5p mimics inhibited cell proliferation, cell cycle progression, and invasion by down-regulating cell-cycle related proteins including cyclin D1, surviving and P53 and down-regulating matrix metallopeptidase 9. In an *in vivo* angiosarcoma xenograft model, TRAM-34 or miR-497-5p mimics both inhibited tumor growth. In conclusion, the tumor suppressor miR-497-5p down-regulates KCa3.1 expression and contributes to the inhibition of angiosarcoma malignancy development. The miR-497-5p or KCa3.1 might be potential new targets for angiosarcoma treatment.

## INTRODUCTION

Angiosarcoma is a malignant neoplasm characterized by infiltrating anaplastic cells derived from vascular endothelial cells. The prognosis of angiosarcoma is poor despite all therapies including radical surgery, adjuvant radiotherapy, and chemotherapy. More than 50% patients die within the first year after diagnosis [1]. It is necessary to understanding the mechanism of angiosarcoma development to evaluate treatments.

Researchers have been working on the genetic aberrations of angiosarcoma to provide insight into the mechanism of tumorigenesis and find effective targets for angiosarcoma molecular therapy. Recurrent driver mutations have been identified in the PTPRB or PLCG1 gene in 38% angiosarcoma patients, including primary and secondary tumors, which reinforce therapeutic efforts to target angiogenesis signaling in angiosarcoma [2]. Secondary angiosarcoma after irradiation or chronic lymphedema may be genetically different from primary angiosarcoma, evidenced by high level of C-MYC amplification [3]. Yang JM, et al provides evidence that disruption of Ink4a/Arf genes in FVB mice is associated with spontaneous angiosarcoma formation by activation of the Ikkβ/NF-κB/IL-6/Stat3 pathway [4].

A class of small RNA molecules known as *microRNAs* (miRNAs or miRs), can negatively regulate gene expression by binding to the 3′-untranslated region (3′-UTR) of target mRNA molecules [5, 6], causing a variety of crucial regulatory functions related to cell growth, development, and differentiation, and are associated with a wide variety of human diseases including cancers [7]. However, limited studies are available about miRNA expression in angiosarcoma. A comprehensive database was developed that contains miRNA expression profiles for 22 types of human sarcomas including angiosarcoma, and 41 miRNAs were identified and exhibited a proximal location in a cluster on chromosome 19 in angiosarcoma compared with adjacent normal tissue [8]. After reverse transcription polymerase chain reaction (RT-PCR) validation, it was proposed that miR-515-3p and miR-517c were tissue specific and potentially may be diagnostic markers for angiosarcoma [8], but the alteration of miRNA expression associated with angiosarcoma malignancy has not been reported.

Potassium channels regulate cancer cell behavior including proliferation and migration, and are associated with channelopathies of cancer. Cancer therapeutic studies that target potassium channels are at an early stage and mostly focused on ether à-go-go (EAG) channels [9]. The KCa3.1, which is a member of the calcium activated potassium channel family, was identified in some cancers including prostate, breast, pancreatic, and endometrial cancers, and is involved in cancer cell proliferation and invasion [10–16]. However, the expression of KCa3.1 has not been identified in any soft tissue sarcomas. The KCa3.1 mRNA is up-regulated in human umbilical endothelial cells in the presence of vascular endothelial growth factor or basic fibroblast growth factor, and required for endothelial cell proliferation and angiogenesis *in vivo* [17, 18]. Up-regulated KCa3.1 also was observed in human endothelial cells of mesenteric arteries from colonic adenocarcinoma patients compared with that in noncancer patients, indicating that KCa3.1 has an altered functional state and possible role in tumor angiogenesis [19].

We wonder whether KCa3.1 and its regulatory miRNAs are expressed and function in angiosarcoma. The purpose of this study was to provide important insight into the molecular alterations relevant to angiosarcoma development and identify potential therapeutic strategies.

## RESULTS

### MicroRNA expression profiles in human angiosarcomas and capillary hemangiomas

**Figure 1:**
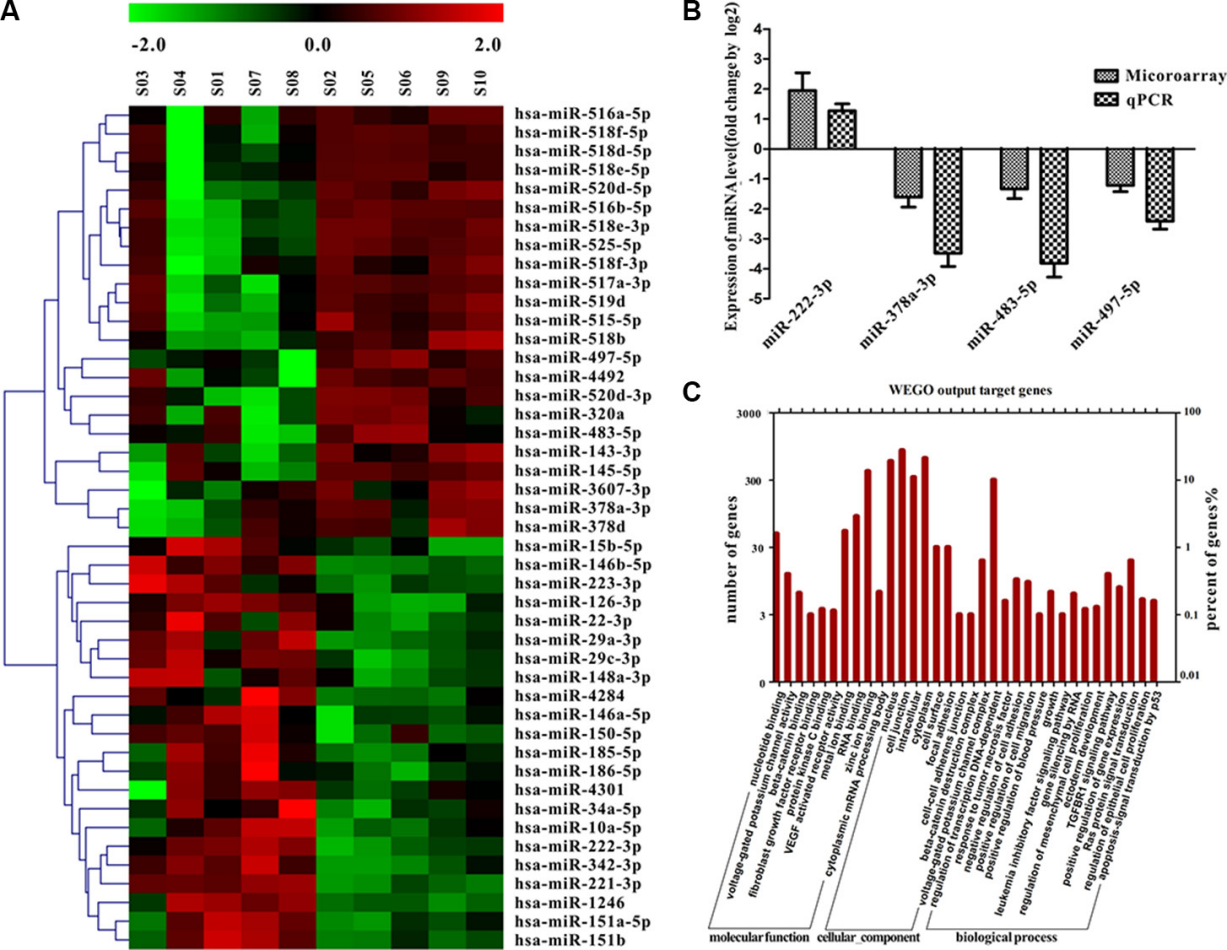
miRNA expression in angiosarcoma and capillary hemangioma and functional annotation of the screened miRNAs (**A**) miRNA expression profiles in 5 angiosarcoma and 5 capillary hemangioma formalin-fixed, paraffin-embedded samples by microarray. (**B**) Five miRNAs are shown according to the relative expression levels by microarray compared with the semiquantitative reverse transcription polymerase chain reaction in 27 angiosarcoma and 15 capillary hemangioma samples. The (log 2)-fold change values are shown on the y-axis. Values are reported as mean ± SE in triplicate (*P* < .01; unpaired *t* test). (**C**) Functional annotations of 4 miRNAs exhibiting similar patterns of dysregulation. Y axis represents the numbers of miRNA-targeted genes that are associated with putative functions.

Expression of miRNA was examined in 5 human angiosarcoma and 5 human capillary hemangioma samples using miRNA array. By comparing miRNA expression profiles, we observed that 45 miRNAs were differentially expressed. Among them, 22 of the 45 miRNAs were up-regulated and 23 miRNAs were down-regulated in angiosarcoma compared with capillary hemangioma (signal intensity > 300, fold of difference > 2, Figure 1A). Among them, 5 selected tumor relevant miRNAs (miR-378-3p, miR-483-5p and miR-497-5p, miR-222-3p and miR-126-3p) were validated with semiquantitative RT-PCR in all 27 angiosarcoma and 15 hemangioma samples. We identified 3 significantly down-regulated miRNAs (miR-378-3p, miR-483-5p and miR-497-5p) and 1 up-regulated miRNA (miR-222-3p) (Figure 1B), which had > 2-fold differences of expression levels between angiosarcoma and hemangioma (Figure 1B).

To explore the biological functions of the differentially expressed miRNAs, the potential targets were evaluated by 3 prediction methods including PicTar, miRDB and miRanda. The genes detected by all 3 independent tools were considered the potential targets of corresponding miRNAs. There were 4 differentially expressed miRNAs predicted to target 2336 mRNAs. We noticed that quite a few potassium channels including KCa3.1 were potential targets of the 4 miRNAs (voltage-dependent potassium channel complex, Figure 1C). We inferred the functions of potential miRNA targets from Gene Ontology (*GO*) term enrichment, including molecular function, cellular component, and biological process. Some significant GO terms were related to cell proliferation or migration. Other GO categories such as regulation of gene expression, protein modification process, and transcription also were significantly enriched (Figure 1C).

### miR-497-5p regulates KCa3.1 expression in angiosarcoma cells

**Figure 2:**
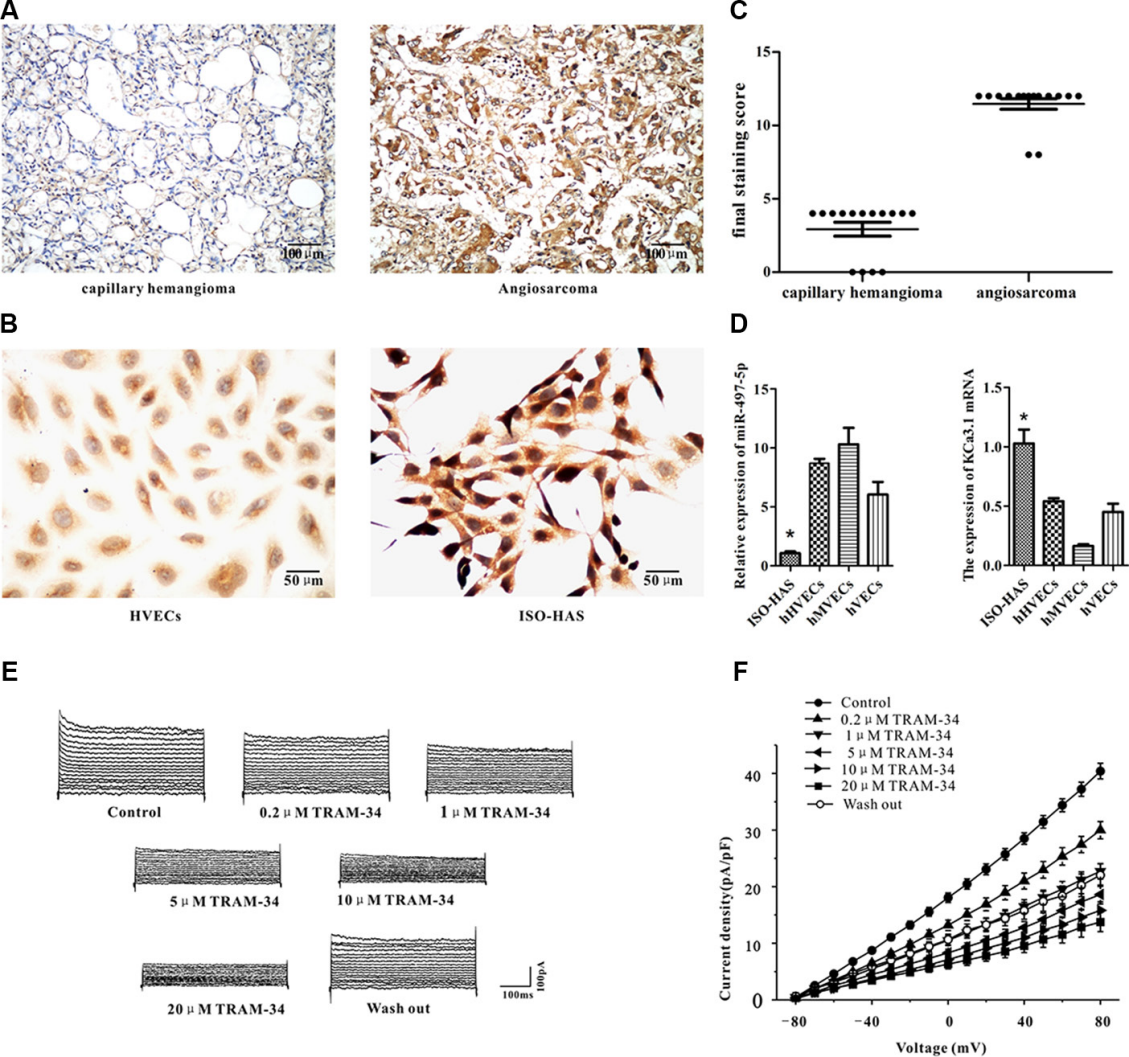
Expression of miR-497-5p and KCa3.1 in angiosarcoma specimens, cell lines, and cultured endothelial cells of varied origin (**A**, **B**) Immunohistochemical staining of KCa3.1 in human capillary hemangioma and angiosarcoma (A) and hHVECs and ISO-HAS cells (B). (**C**) Statistic difference of KCa3.1 expression in human hemangioma and angiosarcoma tissues was demonstrated. (**D**) mRNA expression levels of miR-497-5p (left) and KCa3.1 (right) in ISO-HAS cells, hHVECs, hMVECs, and hVECs by semiquantitative reverse transcription polymerase chain reaction. U6 RNA and GAPDH mRNA were used as normalization controls for miR-497-5p and KCa3.1. Each data point was obtained in triplicate. Data are reported as mean ± SD. (*P < .01; unpaired *t* test). (**E**, **F**) Outward currents of ISO-HAS cells were recorded at different voltage steps from −80 to +80 mV in the presence of different concentration of TRAM-34 (0.2, 1, 5, 10 and 20 μM) (E). Its I/V curve was plotted (F). Abbreviations: hHVECs, human hemangioma endothelial cells; hMVECs, human microvascular endothelial cells; hVECs, human vascular endothelial cells; GAPDH, glyceraldehyde 3-phosphate dehydrogenase.

Among the target genes predicted by bioinformatic software, quite a few potassium channels are listed, and KCa3.1, a potential target gene of miR497-5p, showed highest mRNA expression in human angiosarcoma tissue in our preliminary RT-PCR experiment. By immunohistochemistry, we observed that KCa3.1 was strongly and diffusely expressed in the cytoplasm and membrane of 27 human angiosarcoma samples (93%) (Figure 2A, strongly positive, *n* = 25; moderately positive, *n* = 2). In contrast, no or very weak staining of KCa3.1 was observed in human capillary hemangioma (Figure 2B; weakly positive, *n* = 11; negative, *n* = 4) and vascular endothelial cells of the peritumoral tissue (data not shown). Using 2-level immunohistochemistry grading system, we found that KCa3.1 was highly expressed in human angiosarcoma samples, but not or weakly expressed in human capillary hemangioma samples (Figure 2C).

To determine whether mir-497-5p directly targets KCa3.1 in angiosarcoma, we first studied the expression of miR497-5p and KCa3.1 in tumor cell lines and primary endothelial cells, including ISO-HAS angiosarcoma cells, human hemangioma endothelial cells (hHVECs), human microvascular endothelial cells (hMVECs), and human umbilical vein endothelial cells (hVECs). We observed that expression of miR-497-5p was significantly down-regulated in human ISO-HAS cells compared with HVECs, hMVECs, or hVECs (Figure 2D, left panel). In contrast, much higher expression of KCa3.1 at both protein and mRNA levels were observed in ISO-HAS cells by immunohistochemistry and RT-PCR (Figure 2B; Figure 2D, right panel). Using whole cell patch clamp, KCa3.1 current was measured. TRAM-34, a specific blocker of KCa3.1 current, inhibited the outward currents at different concentration (0.2, 1, 5, 10 and 20 μM) in cultured ISO-HAS cells clamped at different voltage potentials from −80 to 80 mV (Figure 2E). KCa3.1 current was measured as the difference between the currents before and after application of TRAM-34. Current density of KCa3.1 channel gradually decreased with the increase of TRAM-34 concentration (*n* = 5, Figure 2F).

**Figure 3:**
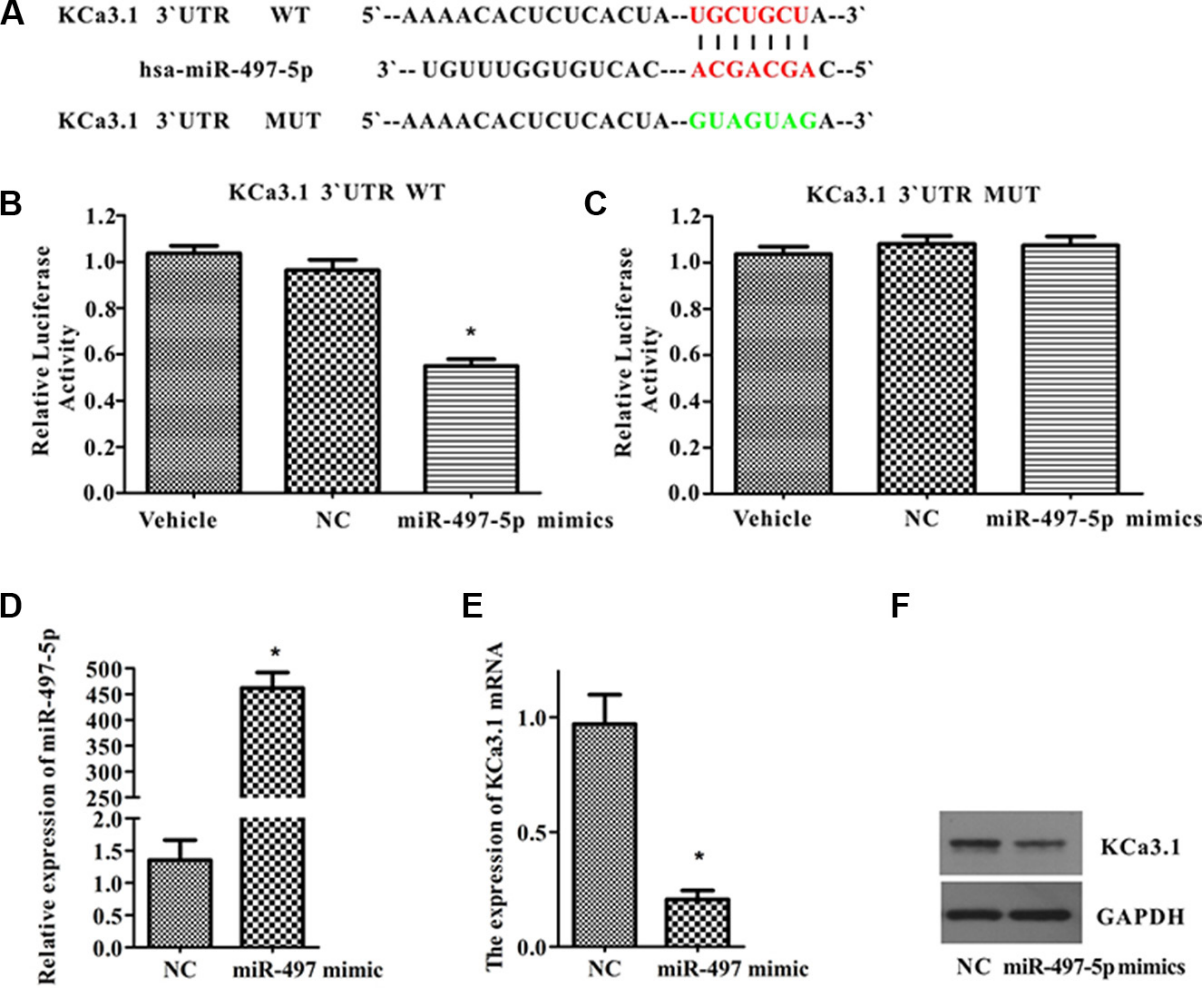
MiR-497-5p targeted KCa3.1 in ISO-HAS cells (**A**) The wild type and mutant complementary sequences of the KCa3.1 mRNA 3′-UTR are shown with the miR-497-5p sequence. (**B**, **C**) Luciferase assay was performed in 293T cells transfected with plasmid containing 3′-UTR KCa3.1 constructs and empty vehicles or non-targeting miR-497-5p (NC) or miR-497-5p mimics (B) or transfected with plasmid containing 3′-UTR Mut KCa3.1 constructs and empty vehicles or non-targeting miR-497-5p (NC) or miR-497-5p mimics (C). Values are the mean ± SE obtained from 3 independent experiments (*P < .01; unpaired *t* test). (**D**) MiR-497-5p expression in ISO-HAS cells transfected with miR-497-5p mimics. (**E**, **F**) Down-regulated KCa3.1 in mRNA (E) and protein levels (F) were observed in ISO-HAS cells transfected with miR-497-5p mimics by semiquantitative reverse transcription polymerase chain reaction and Western blot. Values are reported as mean ± SE obtained from 3 independent experiments (*P < .01 versus control). Abbreviations: GAPDH, glyceraldehyde 3-phosphate dehydrogenase; NC, negative control.

Luciferase reporter assay was performed to verify whether KCa3.1 is the direct target of miR-497-5p. Ectopic expression of miR-497-5p in 293T cells significantly inhibited luciferase activity in the cells transfected with the 3′-UTR KCa3.1 reporter vector, but it did not change luciferase activity in cells transfected with the 3′-UTR Mut KCa3.1 reporter vector (Figure 3A–3C). These results suggested that miR-497-5p was bound complementarily to 3′-UTR KCa3.1, and thus inhibited luciferase activity. This finding was confirmed by the down-regulated expression of KCa3.1 at mRNA and protein levels in ISO-HAS cells transfected with miR-497-5p mimics (Figure 3D–3F). Therefore, we suggested that miR-497-5p negatively regulated the gene expression of KCa3.1 by targeting KCa3.1 mRNA for cleavage.

### Inhibition of KCa3.1 suppressed ISO-HAS cell proliferation *in vitro*

**Figure 4:**
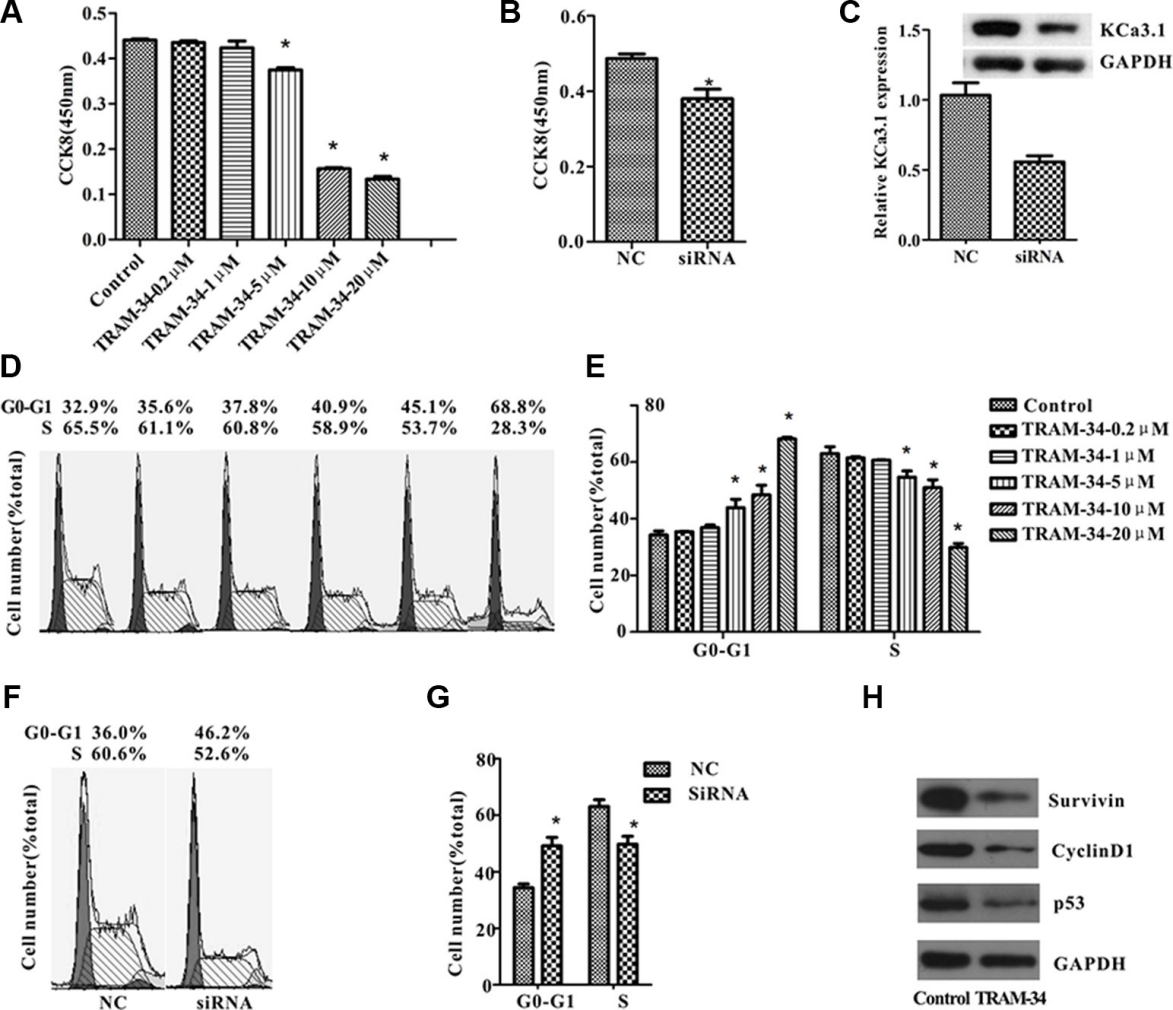
KCa3.1 blockade or knockdown suppressed ISO-HAS cell growth *in vitro* (**A**–**C**) CCK8 assays were performed in ISO-HAS cells after different concentration of TRAM-34 (A) or KCa3.1 siRNA (B) was introduced into the flasks for 24 hours. Transfection of KCa3.1 siRNA suppressed KCa3.1 protein expression for 45% around (C). (**D**–**G**) Cell cycle analysis of ISO-HAS cells was performed after cells were treated with different concentration of TRAM-34 (D, E) or transfected with KCa3.1 siRNA (F, G) for 24 hours. (**H**) Expression of cell cycle related proteins including cyclinD1, surviving and P53 were suppressed in cells treated with 5 μM of TRAM-34. All values are reported as mean ± SE obtained from 3 independent experiments (*P < .01; unpaired *t* test). Abbreviations: TRAM-34, 1-[(2-chlorophenyl) diphenyl-methyl]-1H-pyrazole; NC, negative control.

To investigate whether KCa3.1 is involved in angiosarcoma development, we examined the functional role of KCa3.1 in ISO-HAS cell growth. In our study, TRAM-34 (0, 0.2, 1, 5, 10, or 20 μM) was added to culture dishes that were seeded with the same numbers of ISO-HAS cells [20]. After 24 hours, CCK8 assay showed that TRAM-34 decreased ISO-HAS cell number in a dose-dependent manner, and the 5 μM is the concentration starting to show significance of inhibition (Figure 4A). A similar inhibitory effect was observed when KCa3.1 mRNA was specifically knocked down using siRNA against KCa3.1 (Figure 4B). The protein level of KCa3.1 in cells transfected with the siRNA was 54% ± 6% of that in the cells transfected with nonsilencing RNA (control) (Figure 4C). These results support the hypothesis that KCa3.1 channel is important for angiosarcoma cell growth in culture.

To analyze whether the suppression of ISO-HAS cell proliferation is due to the inhibition of cell cycle progression, the cells treated with TRAM-34 or transfected with KCa3.1 siRNA were examined by flow cytometry. In the presence of TRAM-34 (≥ 5 μM) the percentage of ISO-HAS cells in the G0/G1 phase was significantly increased, but in the S phase was markedly reduced (Figure 4D, 4E). The changes of cell cycle state in the presence of TRAM-34 were concentration dependent. Cells transfected with KCa3.1 siRNA also showed cell cycle arrest at G0/G1 phase (Figure 4F, 4G). Cell cycle-related proteins including cyclinD1, survivin and P53 were downregulated in the presence of 5 μM TRAM-34 (Figure 4H). All above data suggested that block of KCa3.1 channel resulted in cell cycle arrest via down-regulating cell cycle-related proteins.

### Inhibition of KCa3.1 suppressed ISO-HAS cell invasion *in vitro*

**Figure 5:**
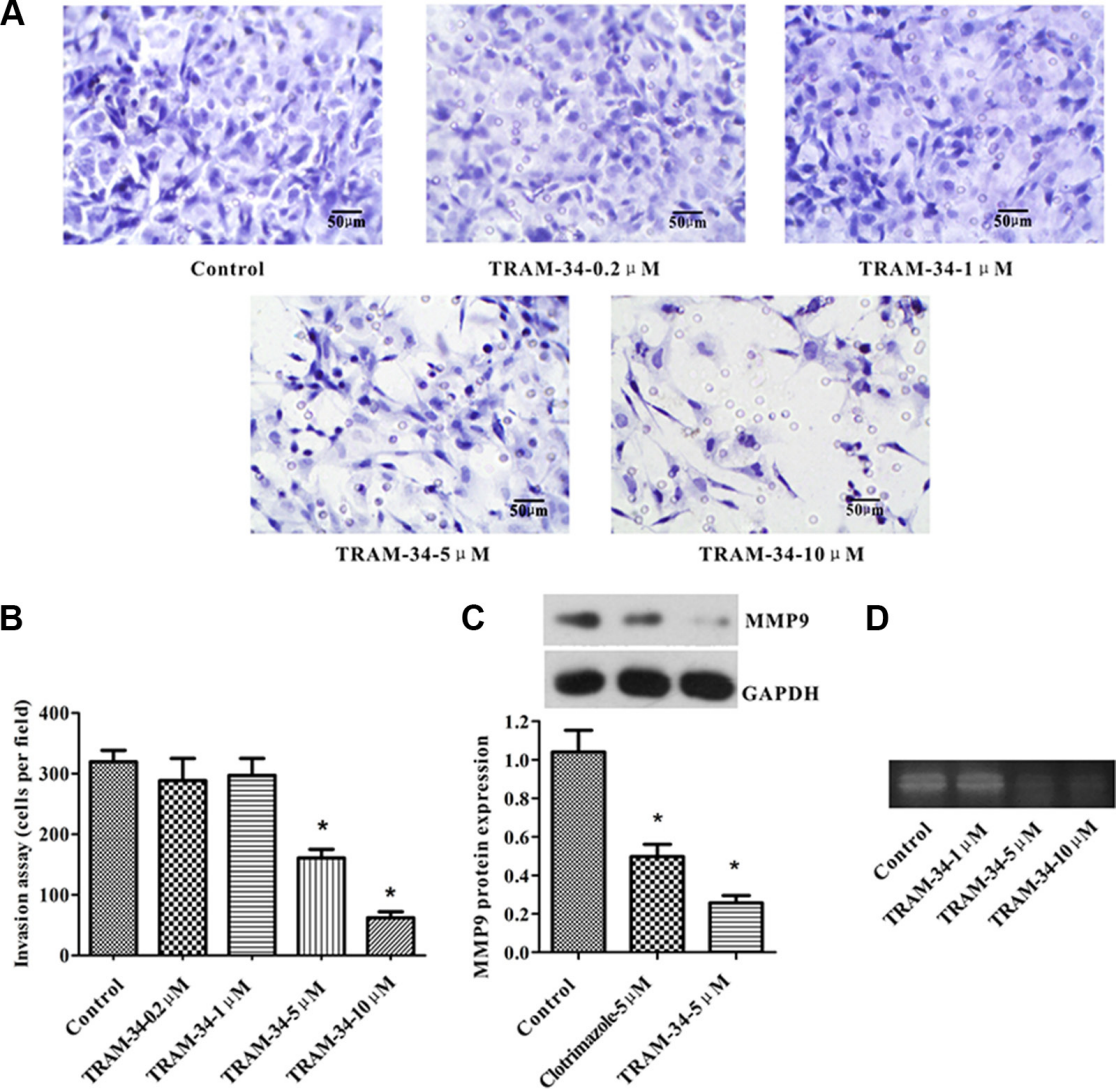
KCa3.1 blockade or knockdown suppressed ISO-HAS invasion *in vitro* (**A**, **B**) Marked decrease of the numbers of invaded cells was observed in the wells treated with 5 or 10 μM TRAM-34. (**C**, **D**) The ISO-HAS cells treated with 5 μM clotrimazole or 5 μM TRAM-34 showed significantly decreased matrix metallopeptidase 9 (MMP9) expression and activity by Western blot and gelatin zymography. Values are reported as mean ± SE from 3 independent experiments (*P < .01; versus control).

The invasiveness of the ISO-HAS cells was examined by invasion assay. TRAM-34 (≥ 5 μM) or clotrimazole (5 μM), another specific blocker of KCa3.1, inhibited ISO-HAS cell invasion through the matrix gel and membrane pores by 80% (Figure 5A–5C). No significant inhibition of cell invasion was observed in the negative control or experimental groups treated with low concentrations of TRAM-34 (0.2 or 1 μM). In addition, Western blot showed that TRAM-34 or clotrimazole suppressed expression of MMP9, a metastasis associated protein (Figure 5C). A decreased MMP9 activity was observed in gelatin zymography in the presence of 5 μM TRAM-34 (Figure 5D). Thus, we propose that the KCa3.1 promoted ISO-HAS cell invasion, which might be associated with the down-regulation of MMP9 protein.

### miR-497 inhibits ISO-HAS cell proliferation and invasion *in vitro* by down-regulation of KCa3.1

**Figure 6:**
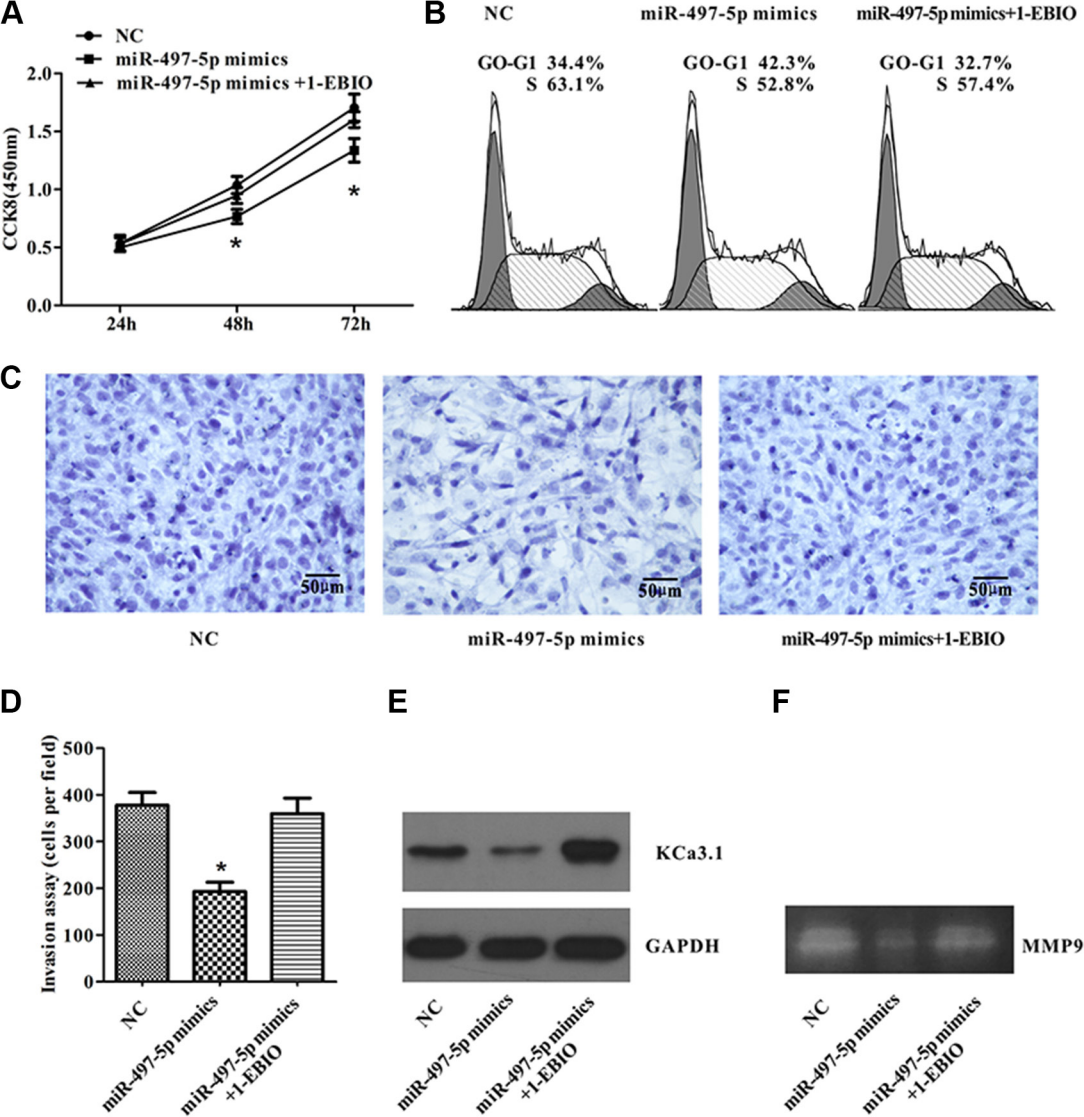
miR-497-5p inhibited ISO-HAS cell proliferation and invasion by down-regulation of KCa3.1 (**A**) Ectopic miR-497-5p expression altered ISO-HAS cell proliferation, which was partially reversed by application of 1-EBIO. (**B**) 1-EBIO partially restored the percentage of cells in M phase after miR-497-5p mimics induced G1 phase arrest. (**C**, **D**) The invasion assay showed that miR-497-5p inhibited ISO-HAS cell invasion, but this effect was reversed by restoration of KCa3.1 with 1-EBIO. (**E**) Western blot assays showed that 1-EBIO induced up-regulation of KCa3.1 channels which has been suppressed by miR-497-5p, Values are reported as mean ± SE (*P < .01; versus control). (**F**) Gelatin zymography showed that a decreased MMP9 activity in miR-497-5p mimic transfected ISO-HAS cells. Abbreviations: 1-EBIO, 1-ethyl-2-benzimidazolinone.

We observed that miR-497-5p targeted KCa3.1, and KCa3.1 played an important role in controlling ISO-HAS cell proliferation and invasion. We explored whether manipulation of miR-497-5p expression can alter tumor cell behavior. After transfecting ISO-HAS cells with miR-497-5p mimics, expression of KCa3.1 was significantly inhibited, and CCK8 assay revealed that the proliferation of ISO-HAS cells overexpressed with miR-497-5p was mildly suppressed by around 20% (Figure 6A). Flow cytometry showed that up-regulation of miR-497-5p retained 10% more ISO-HAS cells in G0/G1 phase and reduced the cell numbers in S phase (Figure 6B). For invasion assay, ISO-HAS cells transfected with miR-497-5p mimics exhibited markedly decreased expression of KCa3.1 and decreased invasive ability by reducing migrated cells for more than 70% (Figure 6C). By application of 500 nM of 1-EBIO, an KCa3.1 channel activator, the inhibition of miR-497-5p mimics on cell proliferation, cell cycle, and cell invasion were partially restored by up-regulating KCa3.1 expression (Figure 6A–6C). A decreased MMP9 activity was observed by gelatin zymography in cells transfected with miR-497-5p mimics (Figure 6F). Therefore, miR-497-5p showed antiproliferative and anti-invasive effects, probably mediated through targeting KCa3.1.

### Blocking KCa3.1 channels or up-regulating miR-497-5p suppressed development of tumors in nude mice

**Figure 7:**
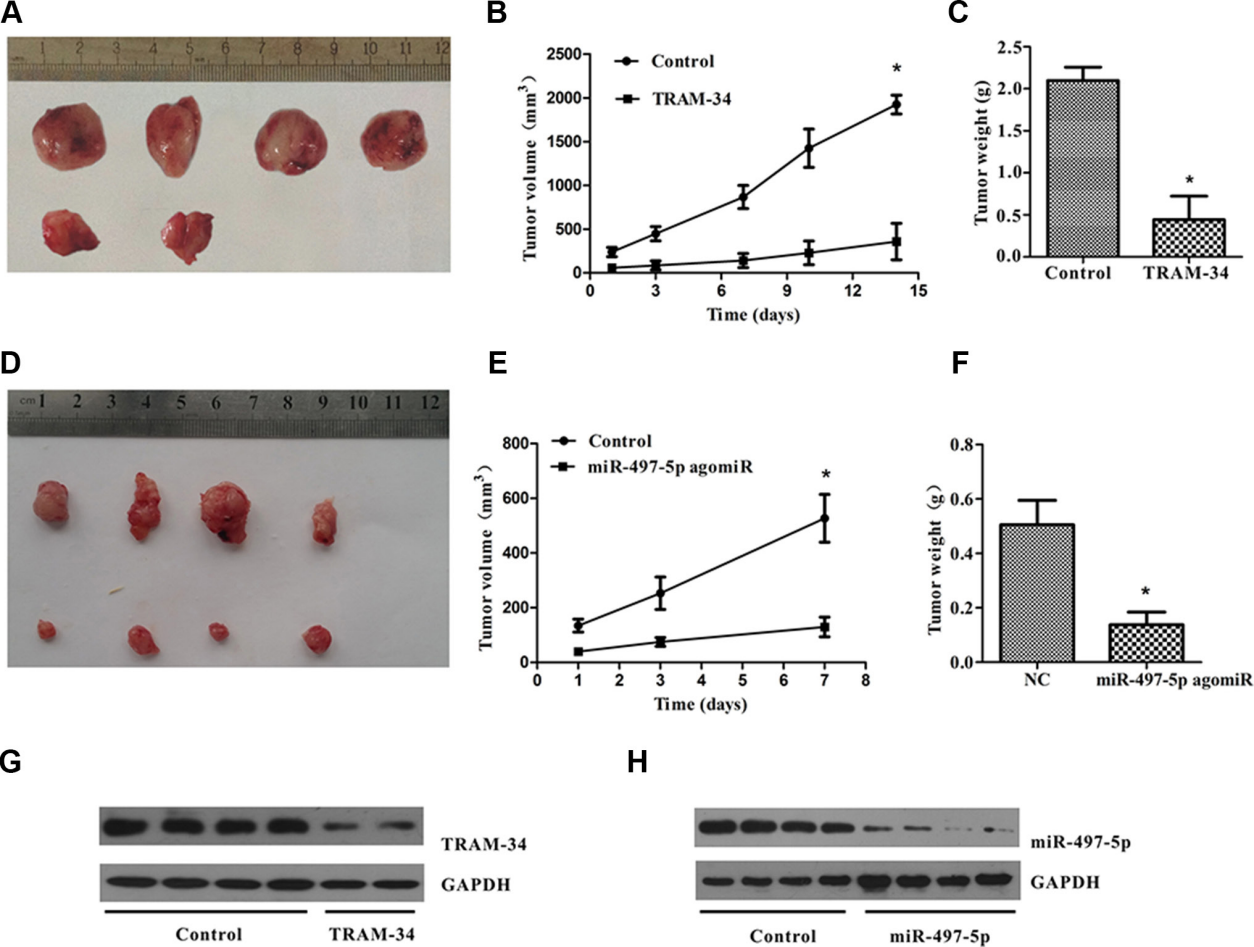
KCa3.1 blockade or miR-497-5p overexpression suppressed the development of angiosarcoma tumors in nude mice (**A**) The ISO-HAS cells (5 × 10^6^) treated with TRAM-34 (final concentration, 30 μM; *n* = 5) or dimethylsulfoxide (control; *n* = 4) were injected into the flank of nude mice. Tumors from TRAM-34-treated (upper row) and control groups (lower row) were removed and photographed 35 days after injection. (**B**, **C**) Volume and weight of xenograft tumors from both groups. (**D**) The miR-497-angomiR (lower row) or nonsense miR-497-angomiR (upper row) overexpressed ISO-HAS cells (5 × 10^6^) were injected into the flank of nude mice. Tumors were removed and photographed 25 days after injection. (**E**, **F**) Volume and weight of xenograft tumors from both groups. Data are reported as mean ± SE. (*P < .01; versus control). (**G**, **H**) The dissected tumors in TRAM-34 and miR-497-5p group showed a significantly decreased KCa3.1 expression as compared with the control groups.

To confirm that KCa3.1 is important for tumor development, we used a BALB/c xenograft mouse model in which mice were inoculated with 5 × 10^6^ ISO-HAS cells treated with or without KCa3.1 inhibitors TRAM-34 (30 μM). Mice were killed at 35 days after injection. All control mice developed visible tumors, with mean tumor weight and volume 2.10 ± 0.1 g and 1931 ± 137 mm^3^ (*n* = 4), but only 40% mice (*n* = 5) in the treated group developed angiosarcoma tumors, with mean tumor weight and volume 0.44 ± 0.28 g and 702 ± 101 mm^3^ (Figure 7A–7C). Similarly, mice inoculated with miR-497-5p transfected cells were killed after 25 days, and xenografted tumors showed markedly decreased weight and size (Figure 7D–7F). The dissected tumors in TRAM-34 and miR-497-5p group showed a significantly decreased KCa3.1 expression as compared with the control group (Figure 7G, 7H). These results suggest that KCa3.1 played an important role in the development of mouse angiosarcoma tumor xenograft *in vivo*.

## DISCUSSION

Advancements in treatment options and improvements in angiosarcoma patient survival have been limited. Targeting the altered expression of genes or miRNAs may be promising therapeutic strategies for angiosarcoma. In the present investigation, we showed that miR-497-5p targeted KCa3.1 in angiosarcoma cells, showing an important role in angiosarcoma cell proliferation and invasion *in vitro* and tumor formation *in vivo*.

In a previous study that profiled miRNA expression of angiosarcoma, miR-515-3p and miR-517c were proposed as diagnostic markers by comparing miRNA expression of angiosarcoma with normal tissues adjacent to tumors and other sarcomas [8]. In the present study, we compared miRNA expression profiles between human angiosarcomas and capillary hemangiomas using miRNA arrays and semiquantitative RT-PCR, and 4 differentially expressed miRNAs might be more relevant to angiosarcoma development. Among these miRNAs, miR-497 is down-regulated in breast, gastric, and colorectal cancer and promotes cell proliferation and invasion by up-regulating raf-1/Ccnd1, EIF4E, and insulin growth factor 1 receptor in breast, gastric, and colorectal cancer [21–23]. In breast cancer, miR-497 expression pattern is negatively correlated with pathologic stage, lymphatic metastasis, tumor size, and HER-2 amplification; therefore, miR-497 may be a new prognostic marker for breast cancer [22]. We observed that miR-497-5p (previously known as miR-497) was down-regulated in angiosarcoma. In addition, forced expression of miR-497-5p suppressed angiosarcoma cell proliferation, blocked cell cycle progression, and reduced cell invasion (Figure 6). This suggests that miR-497-5p is a suppressor of angiosarcoma, as reported in other cancers.

We predicted and confirmed that KCa3.1 is a novel target of miR497-5p (Figure 3). It is known that elevated intracellular calcium activates KCa3.1 and maintains a negative membrane potential, which helps to sustain calcium entry into the cell and modulate calcium signaling processes [24]. Sustained calcium entry due to activation of KCa3.1 is important for the production of inflammatory chemokines by inflammatory cells [25, 26] and proliferation of lymphocytes and fibroblasts [26]. In addition, KCa3.1 is overexpressed in a variety of human tumor cell lines and human biopsy samples including glioblastoma [30–32], pancreatic cancer [12], prostate cancer [33], endometrial cancer [16] and breast cancer [15, 34]. KCa3.1 channels contribute to tumor cell proliferation by promoting cell cycle transition from G0/G1 to S phase [10, 12–16]. In this study, we showed that KCa3.1 was up-regulated in human biopsies and a cell line of angiosarcoma (Figure 2). Consistent with findings in other cancer cells, suppression of KCa3.1 by application of TRAM-34 or siRNA transfection or up-regulating miR-497-5p strongly inhibited angiosarcoma cell proliferation by down-regulating cyclinD1, survivin and P53 and retaining cells in G0/G1 phase (Figure 4). Membrane potential change is important for G1/S phase and G2/M phase transitions, and hyperpolarization is required for S phase initiation [35]. Therefore, a reasonable explanation for the fact that KCa3.1 promotes angiosarcoma cell proliferation is that up-regulated KCa3.1 hyperpolarizes the membrane potential and induces more cells entering into S phase.

In addition to the functional role in cell proliferation, miR-497-5p/KCa3.1 also is important for cell invasion. The KCa3.1 channels are involved in regulating glioblastoma cell migration *in vitro* in response to CXCL12 [36] and bradykinin [37]. The importance of KCa3.1 for glioblastoma invasion has been further supported by the observations that KCa3.1 channel inhibition with TRAM-34 reduced glioblastoma infiltration in the brain parenchyma in response to tumor-released factors [11]. In this study, we showed that KCa3.1 is important for angiosarcoma cell invasion because inhibiting KCa3.1 channel with TRAM-34 or up-regulating miR-497-5p reduced the cell migration through a matrix gel (Figure 5). Such inhibition on angiosarcoma cell invasion may be due to decreased protein expression of MMP9 and reduced hydrolysis of extracellular matrix (Figure 5). However, the other 2 mechanisms (cell volume regulation or control of the calcium influx) through which KCa3.1 contributes to glioblastoma cell migration also may apply to angiosarcoma cell migration [38].

We have suggested that miR-497-5p targeted KCa3.1 and exhibited a tumor suppressive effect by inhibiting angiosarcoma cell proliferation and invasion. We observed that inducing miR-497-5p mimics into angiosarcoma cells resulted in strong inhibition of cell invasion, but only mild reduction of cell proliferation, which is different from the inhibitory effects of TRAM-34 on proliferation and invasion of angiosarcoma cells. A possible explanation for this observation is that KCa3.1 channels might be regulated by other molecules or signal pathways that have not yet been identified, or miR-497-5p may have other targets that are involved in tumor malignancy. However, in the *in vivo* experiment, miR-497-5p strongly inhibited tumor development similar to TRAM-34, possibly because miR-497-5p may have restricted tumor development by strongly inhibiting tumor cell invasion to the surrounding tissue and decreasing cell proliferation.

Researchers have been looking for effective therapies. Quite a few clinical trials have been performed including sorafenib targeting Raf/MAPK pathway [39], imatinib targeting BCR-ABL/VEGF pathway [40] and some chemotherapeutic agents (doxorubicin and taxane) [41, 42] in patients with advanced or metastatic angiosarcoma, however, only limited effect was obtained. The present study provided evidence that miR-497-5p targeting KCa3.1 is strongly associated with malignancy development of human angiosarcoma. Thus the miR-497-5p/KCa3.1 pair would be a new promising potential target for the treatment of angiosarcoma.

## MATERIALS AND METHODS

### Patients

Tissues from angiosarcoma (27 patients) and capillary hemangioma (15 patients) were obtained from pathology departments at hospitals in Hubei Province, China. All tissue samples were fixed in formalin and embedded in paraffin. Tissue slides were independently reviewed by 2 pathologists and diagnosed as primary angiosarcoma. Their clinical information was shown in Supplementary Table S1. The ethics committee of Tongji Hospital approved this study.

### Cell culture and drugs

The phenotypic angiosarcoma cell lines ISOS-1 (murine) and ISO-HAS (human) were provided by Dr. Mikio Masuzawa (Kitasato University, Kanagawa, Japan). Up to now, there are only two available angiosarcoma cell lines (ISO-HAS and ASM). We were unable to obtain the human angiosarcoma ASM cell line; therefore, the experiments on human angiosarcoma cells were limited to ISO-HAS cells. The ISOS-1 and ISO-HAS cells were cultured as described previously [43]. Briefly, ISOS-1 cells were cultured in complete medium, which was high-glucose Dulbecco modified Eagle medium (DMEM, Gibco BRL, Gaithersburg, MD, USA) supplemented with 15% fetal bovine serum (FBS) (Gibco, 10099-141, Australia), and ISO-HAS cells were cultured in 50% complete medium and 50% ISOS-1 conditioned medium. The ISOS-1 conditioned medium was obtained after ISOS-1 cells were cultured in complete medium for 3 to 4 days and filtered (0.22-μm bottle top filter). Human hemangioma endothelial cells (hHVECs), human microvascular endothelial cells (hMVECs), and human umbilical vein endothelial cells (hVECs) grew in conventional culture media.

1-[(2-chlorophenyl)diphenyl-methyl]-1H-pyrazole (TRAM-34), 1-ethyl-2-benzimidazolinone (1-EBIO), and clotrimazole were purchased (Sigma, St. Louis, MO, USA), dissolved in dimethylsulfoxide (DMSO) with a stock concentration of 10 mM, and stored at −20°C. The KCa3.1 (ab83740) and matrix metallopeptidase 9 (MMP9, ab76003) antibodies were purchased (Abcam, Cambridge, UK).

### RNA extraction

Total RNA from formalin-fixed and paraffin-embedded (FFPE) human angiosarcoma (*n* = 27) and capillary hemangioma (*n* = 15) was extracted using a kit (RecoverAll Total Nucleic Acid Isolation Kit, Ambion, TX, USA) according to instructions from the manufacturer. RNA from cultured cells was isolated (Trizol reagent, Invitrogen, Carlsbad, CA, USA). RNAs were quantified with a spectrophotometer (NanoDrop ND-1000, Thermo Fisher Scientific, Wilmington, UK) and qualified by electrophoresis on a denaturing agarose gel.

### Microarray hybridization and data analysis

Equal amounts of RNAs were pooled, and microarray assay and analysis (miRBase 18) were performed by a service provider (LC Sciences, Houston, TX, USA). Normalization was performed using a cyclic locally weighted scatterplot smoothing (LOWESS) regression. Differential expression was analyzed using a 2-group *t* test to determine the miRNAs that were differentially expressed between the angiosarcoma and hemangioma groups. The data with *P* < .05 were selected for cluster analysis.

### Semiquantitative RT-PCR

Semiquantitative RT-PCR was performed to verify the expression levels of miRNAs and mRNAs in human tumor tissues and cultured tumor cells. Total RNAs (1 μg) were reverse transcribed using Moloney Murine Leukemia Virus (M-MLV) reverse transcriptase with oligodeoxythymine (oligo-dT) for KCa3.1 mRNAs (gene bank accession no., NM 002250-3) or with special stem-loop primers (5′-GTCGTATCCAGTG CAGGGTCCGAGGTATTCGCACTGGATACGACACA AAC-3′) for miR497-5p (MiRbase, MIMAT0002820). Semiquantitative RT-PCR was performed (StepOnePlus Real-Time PCR Systems, Applied Biosystems International, Inc., Delaware, USA) using a dye (SYBR Green, Qiagen, Shanghai, China) to amplify KCa3.1 (sense, 5′-GAGAGGCAGGCTGTTAATGC-3′; antisense, 5′-ACGTGCTTCTCTGCCTTGTT-3′) and miR497-5p (sense, 5′-CCTTCAGCAGCACACTGTGG-3′; antisense, 5′-CAGTGCAGGGTCCGAGGTAT-3′). The relative expression levels of miR497-5p and KCa3.1 mRNAs were normalized to human U6 small nuclear RNA and human glyceraldehyde-3-phosphate dehydrogenase (GAPDH), and expressed as mean ± standard deviation (SD).

### Western blot

Cells were washed with phosphate-buffered saline (PBS) and lysed in extraction buffer (Thermo Fisher Scientific Pierce, Rockford, IL, USA). Protein (25 μg) from each sample was separated on 12% sodium dodecyl sulfate polyacrylamide gel electrophoresis (SDS–PAGE) and transferred to a nitrocellulose membrane. Immune complexes were formed by incubation of proteins with primary antibodies overnight at 4°C. The membrane was incubated with horseradish peroxidase-conjugated secondary antibodies and visualized by ECL (Thermo Pierce, Cramlington, UK). Images were captured (Kodak Image). The intensity of the immunoreactive band was normalized to the intensity of β-actin band.

### siRNA transfection

The siRNA against KCa3.1 (NM_002250-3) (5-GCCGUGCGUGCAGGAUUUA-3 [sense] and 5-UAAAUCCUGCACGCACGGC-3 [antisense]) and nonsilencing siRNA (5-UUCUCCGAACGUGUCACGU-3 [sense]; 5-ACGUGACACGUUCGGAGAA-3 [antisense]) were synthesized by a service provider (GenePharma Co., Ltd, Guangzhou, China). The ISO-HAS cells were transfected with siRNAs using a transfection reagent (Lipofectamine 2000, Invitrogen, Carlsbad, CA, USA) according to the protocol from the manufacturer. The efficiency of siRNA to knock down the target protein was determined by Western blot.

### Luciferase assay

For luciferase assay, the 3′-UTR of KCa3.1 (NM_002250-3UTR) containing the miRNA-497-5p binding sites and its corresponding mutated sequence were cloned into pRL-TK luciferase reporter vector (Promega, Madison, WI, USA), named 3′-UTR KCa3.1 and 3′-UTR Mut KCa3.1 constructs. Using the transfection reagent (Lipofectamine 2000, Invitrogen) plasmid expressing 3′-UTR KCa3.1 or 3′-UTR Mut KCa3.1 constructs and miR-497-5p mimics (Ribo, Guangzhou, China) or non-targeting miR-497-5p (Ribo, Guangzhou, China) or empty vehicles were cotransfected into cultured 293T cells. Cells treated with transfection reagent with empty vectors and miR-497-5p mimics were the negative control. Luciferase activity was measured after 48 h using a kit (Dual-Glo Luciferase Assay kit, Promega). Data were presented as the ratio of experimental (Renilla) luciferase to control (firefly) luciferase.

### Cell proliferation assay and cell cycle analysis

TRAM-34 was added (final concentration: 0, 0.2, 1, 5, 10, or 20 μM) to the ISO-HAS cell culture flasks. Wells containing 0.1% DMSO or culture medium were established as controls. Cell proliferation was assessed using a kit (Cell Counting Kit-8 [CCK8], Dojindo, Kumamoto, Japan) according to the instructions from the manufacturer. Cells were seeded on a 96-well plate (Corning Inc., Corning, NY, USA) at 1 × 10^4^/well in volume 100 μL and cultured for 24 hours. The kit (CCK8) reagents were added to the wells (10 μL/well). After incubation (2 h), absorbance of the cells was read on an automated plate reader (SmartSpec Plus, Bio-Rad Hercules, CA, USA) at wavelength 450 nm.

The ISO-HAS cells were treated with TRAM-34 at different concentrations (0, 0.2, 1, 5, 10, or 20 mM) for 24 hours and fixed with 75% ethanol. Cellular DNA was labeled by propidium iodide staining of detergent-permeabilized cells at 4°C for 30 minutes. Cell cycle status was measured by flow cytometry (Becton Dickinson, North Ride, NSW, Australia) and analyzed using software (ModFit LT, version 2, Verity Software House, Topsham, ME, USA). Cell proliferation and cell cycle were assessed on the miR-497 mimic transfected cells with or without subsequent addition of KCa3.1 channel activator 1-EBIO.

### Invasion assay

Cells were pretreated with TRAM-34 at different concentrations (0, 0.2, 1, 5 and 10 μM) for 8 hours or transfected with miR-497-5p mimics with or without subsequent addition of 500 nM 1-EBIO. Invasion assay was performed using 8.0-nm pore size inserts (Transwell, Corning) that were coated with matrix (Matri-gel, Corning). A total 1.5 × 10^5^ cells were suspended in 200 μL serum-free medium and loaded in the upper compartment of the insert, which was sitting in a well containing 500 μL culture medium supplemented with 10% FBS. After 36 hours the invaded cells on the underside of the inserts were fixed with 1% paraformaldehyde for 5 minutes, washed with PBS for 1 minute, stained with hematoxylin for 3 minutes, and then counted. There were 2 repetitions performed for each group.

### Zymography assay

Activities of MMP-9 in ISO-HAS cells were determined by gelatin zymography. In details, ISO-HAS cells in serum-free medium containing 0.1% DMSO or TRAM-34 (1, 5 or 10 μM) or cells transfected with miR-497 mimics were seeded in 24-well plates with a density of 2 × 10^5^ cells/well, and incubated for 24 h. Electrophoresis was performed on a 10% polyacrylamide/SDS gel containing 1 mg/mL gelatin. The gel was washed with 2.5% Triton X-100 for 1 h and incubated overnight at 37°C in enzyme buffer (50 mM Tris, pH 7.5, 200 mM NaCl, 5 mM CaCl_2_ and 0.02% Brij-35). Areas of gelatin hydrolyzed by MMP were visualized as clear zones against blue background.

### Immunohistochemistry

Immunohistochemistry was performed on 4-μm, formalin-fixed, paraffin-embedded sections with anti-KCa3.1. Briefly, the slides were deparaffinized, rehydrated, and dripped in 3% hydrogen peroxide solution for 10 minutes. The slides were incubated with anti-KCa3.1 (1:150 dilution) at 4°C overnight. After washing with PBS, the slides were incubated with Polymer Helper for 20 minutes and polyperoxidase-antihuman immunoglobulin G for 30 minutes at room temperature. Slides were developed with a kit (EnVision Detection Kit, Maxim Biotect, Fuzhou China). Hematoxylin was used as counterstain, and the negative control was obtained by substituting the primary antibody with PBS. Slides were examined by 2 pathologists (Tongji Hospital, China) blindly and independently. Immunohistochemical staining was evaluated as multiplication of the percentage score of positively stained tumor cells and the staining intensity score. The intensity score was defined as: 0 (negative), 1 (weakly positive), 2 (moderately positive), and 3 (strongly positive). The percentage score was defined as 0 (negative), 1 (< 10%), 2 (11% to 50%), 3 (51% to 80%) and 4 (> 80% positive cells). The degree of KCa3.1 staining was quantified using a 2-level grading system as follows: < 6 indicates low expression, while 6 to 12 indicates high expression.

### Electrophysiology

The whole-cell patch-clamp technique was used [44]. Patch pipettes were made from borosilicate fiber-containing glass (Clark Electromedical Instruments, Reading, UK) and the tips were heat-polished, resulting in resistance 3-4 MΩ. The pipette solution contained 130 mM potassium chloride, 5 mM magnesium chloride, 1 mM calcium chloride, 10 mM 4-(2-hydroxyethyl)-1-piperazineethanesulfonic acid (HEPES), and 3 mM ethylene glycol tetraacetic acid (pH, 7.3). The external solution contained 136 mM sodium chloride, 5.4 mM potassium chloride, 1.0 mM magnesium chloride, 1.8 mM calcium chloride, 0.33 mM monosodium phosphate, 10 mM glucose, and 10 mM HEPES (pH, 7.3). Whole cell currents were recorded with an amplifier (Axoclamp 200A, Axon Instruments, CA, USA), and currents were evoked by applying voltage commands to a range of potentials (−80 to + 80 mV) in 10 mV steps from a holding potential of −80 mV. The data were digitalized (sampled at 10 kHz), stored on a computer, and analyzed using software (pClamp, Axon Instruments). Data were presented as mean ± standard error (SE). Statistical significance was determined by *t* test, and *P* < .05 was considered significant.

### Tumor xenograft model

BALB/c nude mice (nu/nu; 6- to 8-week-old females) were obtained from the animal facility of Tongji Hospital and maintained under clean conditions. Care and handling of the animals were in accordance with the Guidelines for Animal Care and Use Committee of Tongji Hospital. Animals were inoculated subcutaneously in the right flank with 5 × 10^6^ ISO-HAS cells suspended in 200 μL PBS containing 0.1% DMSO (TRAM-34 control group, 4 mice) or 30 μM TRAM-34 (TRAM-34 group, 5 mice), or transfected with miR-497-agomiR (miR-497-5p group, 4 mice) or non-targeting-miR-497-agomiR (miR-497-5p negative control group, 4 mice). Nude mice were sacrificed with carbon dioxide inhalation 25 or 35 days after cell inoculation. All xenograft tumors were dissected, photographed, and weighed, and volume V was calculated using the following formula: V = 0.5 × L × W^2^, where L was length and W was width.

## SUPPLEMENTARY MATERIALS FIGURES AND TABLE


