# Supplementary material for: miR-497-5p inhibits cell proliferation and invasion by targeting KCa3.1 in angiosarcoma
Source: Oncotarget. 2016 Aug 12;7(36):58148–61. doi: 10.18632/oncotarget.11252 (PMC5295420; doi:10.18632/oncotarget.11252)
Supplement: Supplementary file 1 [file oncotarget-07-58148-s001.pdf]

miR-497-5p inhibits cell proliferation and invasion by targeting KCa3.1 in angiosarcoma

Supplementary Materials

A

The siRNAs targeting KCa3.1 (KCNN4) (NM_002250-3).

siR-1 5-GCCGUGCGUGCAGGAUUUA-3 [sense]
5-UAAAUCCUGCACGCACGGC-3 [antisense]

siR-2 5-CUGGUUAAAUGCACGAUCA-3[sense]
5-UGAUCGUGCAUUUAACCAG-3[antisense]

siR-3 5-CAUGAUGGAUAUCCAGUAU-3[sense]
5-AUACUGGAUAUCCAUCAUG-3[antisense]

Non-silencing siRNA
5-UUCUCCGAACGUGUCACGU-3[sense]
5-ACGUGACACGUUCGGAGAA -3[antisense]

B

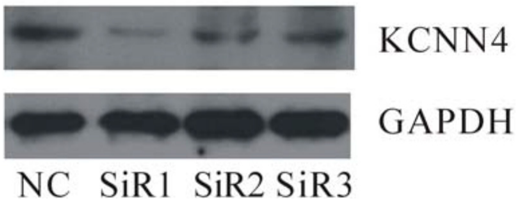

Supplementary Figure S1: (A) Knockdown KCa3.1 expression by three designed siRNAs. (**B**) Westernblot showed KCa3.1 expression in ISO-HAS cells transfected with SiR-1, SiR-2, SiR-3 and vehicle (negative control, NC).

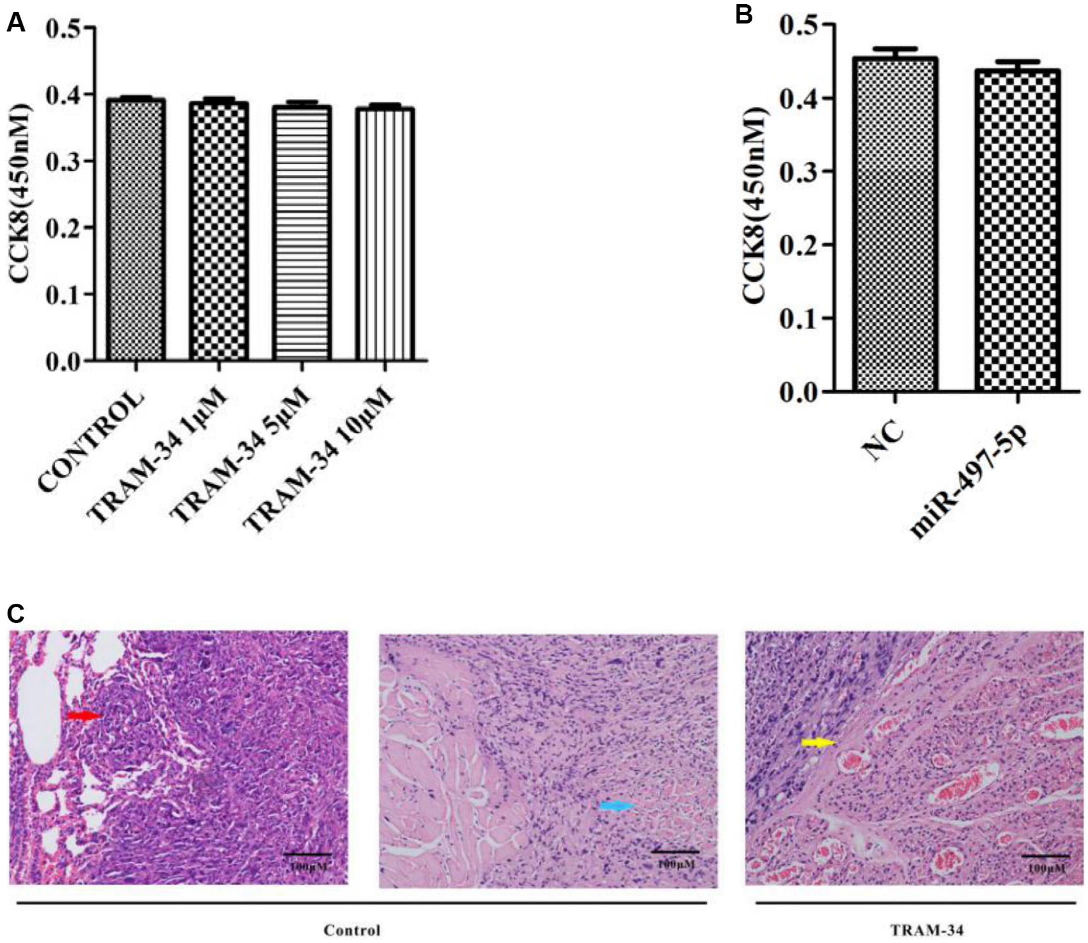

Supplementary Figure S2: Reduction of invaded tumor cells is not due to the inhibited proliferation of TRAM-34 treated or miR-497-5p mimic transfected cells. (**A**) TRAM-34 (1 μM, 5 μM or 10 μM) pretreated ISO-HAS cells showed no significant difference of cell viability by CCK8 assay in serum free condition for 36 hours. (**B**) MiR-497-5p mimic transfected or untransfected ISO-HAS cells showed no significant difference of cell viability by CCK8 assay in serum free condition for 36 hours. (**C)** TRAM-34 inhibited tumor cell invasion in tumor xenograft model. Infiltrative invasion to lung (left, $n = 1$) and skeletal muscle (middle, $n = 2$) was observed in TRAM-34 control group ($n = 4$). However, tumors from TRAM-34 group (right, $n = 2$) showed less infiltrative boundary.

Supplementary Table S1: Clinical data of angiosarcoma and capillary hemangiomas

	Case NO.	Age	Gender	Location	Differentiation
**Angiosarcoma**	Case1	23	female	breast	high
	Case2*	61	male	Submandibular gland	low
	Case3	41	male	Mediastinum	middle
	Case4	33	male	Mediastinum	middle
	Case5	38	male	sternum	middle
	Case6*	48	female	retroperitoneum	middle
	Case7	13	male	Shoulder skin	low
	Case8*	65	female	liver	middle
	Case9*	47	female	spleen	high
	Case10	51	male	Parapharyngeal	middle
	Case11	62	female	heart	high
	Case12	73	male	scalp	low
	Case13	51	female	thigh	middle
	Case14	66	female	neck	low
	Case15	72	male	retroperitoneum	low
	Case16	35	female	heart	low
	Case17	71	male	scalp	middle
	Case18	54	male	femur	low
	Case19	27	female	retroperitoneum	middle
	Case20	59	female	heart	middle
	Case21*	63	female	ilium	middle
	Case22	56	male	mediastinum	low
	Case23	27	female	ear	low
	Case24	44	male	heart	middle
	Case25	54	female	retroperitoneum	high
	Case26	39	male	retroperitoneum	middle
	Case27	70	female	vulva	high

**Capillary hemangiomas**	Case NO.	Age	Gender	Location	
	Case1	3 month	female	scalp	
	Case2	5 month	female	abdomen	
	Case3	6 month	female	back	
	Case4*	3 month	female	shoulder	
	Case5	16 month	male	shoulder	
	Case6*	6 month	female	neck	
	Case7*	4 month	male	back	
	Case8*	8	female	back	
	Case9*	4 month	female	neck	
	Case10	46	female	epiglottis	
	Case11	8	female	neck	
	Case12	52	male	forehead	
	Case13	3	male	leg	
	Case14	4	female	labia	
	Case15	2	female	abdominal	
